# Mechanical Characteristics and Self-Healing Soil-Cementitious Hydrogel Materials in Mine Backfill Using Hybridized ANFIS-SVM

**DOI:** 10.3390/gels8070455

**Published:** 2022-07-21

**Authors:** Qi Liu, Kang Peng, Yousef Zandi, Alireza Sadighi Agdas, Haneen M. Al-Tamimi, Hamid Assilzadeh, Ahmed Abdel Khalek Ebid, Mohamed Amine Khadimallah, H. Elhosiny Ali

**Affiliations:** 1 School of Resources and Safety Engineering, Central South University, Changsha 410083, China; weihun1@hotmail.com; 2 Changsha Institute of Mining Research Co., Ltd., Changsha 410083, China; 3 Department of Civil Engineering, Tabriz Branch, Islamic Azad University, Tabriz 51579, Iran; zandi@iaut.ac.ir; 4 Ghateh Gostar Novin Company, Tabriz 51579, Iran; alireza.sadighi.agdas@gmail.com; 5 Air Conditioning and Refrigeration Techniques Engineering Department, Al-Mustaqbal University College, Babylon 51001, Iraq; haneen.mohammed.hadi@mustaqbal-college.edu.iq; 6 Department of Biomaterials, Saveetha Dental College and Hospital, Saveetha Institute of Medical and Technical Sciences, Chennai 600077, India; hamidassilzadeh@aol.com; 7 Structural Engineering and Construction Management, Faculty of Engineering, Future University in Egypt, New Cairo 11745, Egypt; ahmed.abdelkhaleq@fue.edu.eg; 8 Department of Civil Engineering, College of Engineering, Prince Sattam bin Abdulaziz University, Al-Kharj 16273, Saudi Arabia; m.khadimallah@psau.edu.sa; 9 Laboratory of Systems and Applied Mechanics, Polytechnic School of Tunisia, University of Carthage, Tunis 1054, Tunisia; 10 Advanced Functional Materials & Optoelectronic Laboratory (AFMOL), Department of Physics, Faculty of Science, King Khalid University, Abha 61413, Saudi Arabia; hibrahim@kku.edu.sa; 11 Research Center for Advanced Materials Science (RCAMS), King Khalid University, Abha 61413, Saudi Arabia; 12 Physics Department, Faculty of Science, Zagazig University, Zagazig 44519, Egypt

**Keywords:** self-healing, soil, cement, hydrogel materials, mechanical strength, autogenous shrinkage, SVM, ANFIS

## Abstract

The compressive strength, shrinkage, elasticity, and electrical resistivity of the cement-soil pastes (slag, fly ash) of self-healing of cementitious concrete have been studied while adding hydrogels with nano silica (NSi) in this research. Defining the hydraulic and mechanical properties of these materials requires improvement to motivate more uptake for new buildings. Initially, examining the impact of different synthesized hydrogels on cement-soil pastes showed that solid particles in the mixtures highly affected the absorption capacity of NSi, representing the importance of direct interactions between solid particles and hydrogels in a cementitious matrix. All test results were analyzed by use of a hybridized soft computing model such as the adaptive neuro fuzzy inference system (ANFIS) and support vector regression (SVR) for precise studying and the avoidance of few empirical tests or error percentages. Subsequently, the best RMSE of ANFIS is 0.6568 and the best RMSE of SVM is 1.2564; the RMSE of ANFIS-SVM (0.5643) in the test phase is also close to zero, showing a better performance in hypothesizing self-healing soil-cementitious hydrogel materials in mine backfill. The R2 value for ANFIS-SVM is 0.9547, proving that it is a proper model for predicting the study’s goal. Electrical resistivity and compressive strength declined in the cement-soil pastes including hydrogels according to experimental outcomes; it was lowered by the increase of NSi concentration in the hydrogel. There was a decrement in the autogenous shrinkage of cement-soil pastes while adding hydrogel, depending on the NSi concentration in the hydrogels. The findings of this research are pivotal for the internal curing of cementitious materials to define the absorption of hydrogels.

## 1. Introduction

Cement is one of the most commercialized materials worldwide [1,2]. It is estimated that global cement consumption was 4.08 billion tons in 2019 [3]. Its use is essential in the manufacture of concrete, the construction phase, and the maintenance of buildings. From 5 to 8% of global CO_2_ emissions correspond to the cement industry due to the calcination of limestone and the combustion of fossil fuels [4]. Additionally, a large electricity supply is required for the limestone-, clinker-, and cement-crushing processes. Cement production consumes 7% of the global industrial energy. Cement production also causes other environmental impacts in addition to greenhouse gas emissions [5]; for example, nitrogen oxides (NO_x_) and volatile organic compounds (VOC), which can produce photochemical ozone formation in the air. This causes health problems [6] and also affects ecosystems [7]. Soil exposure to nitrogen oxides (NO_x_), ammonia (NH_3_), and sulfur dioxide (SO_2_) in high concentrations causes acidification, which can cause problems in the growth and development of vegetation. Concrete is a widely applied material in construction, and it is composed mainly of cement, crushed stone, sand, and water. The combination, proportion, number of components, and additions of these raw materials will result in the final properties of the concrete [8,9]. The versatility in its manufacturing is that is can be produced on-site or ready-mixed, and it is easy to mold into different shapes and sizes. This, combined with its mechanical properties, durability, chemical inertness, thermal energy storage, and cost make it an essential material in the construction sector. It is the most-consumed material after water. This extensive use makes evaluating and analyzing its environmental impacts essential, considering concrete production and its impact on climate change.

**Figure 1:**
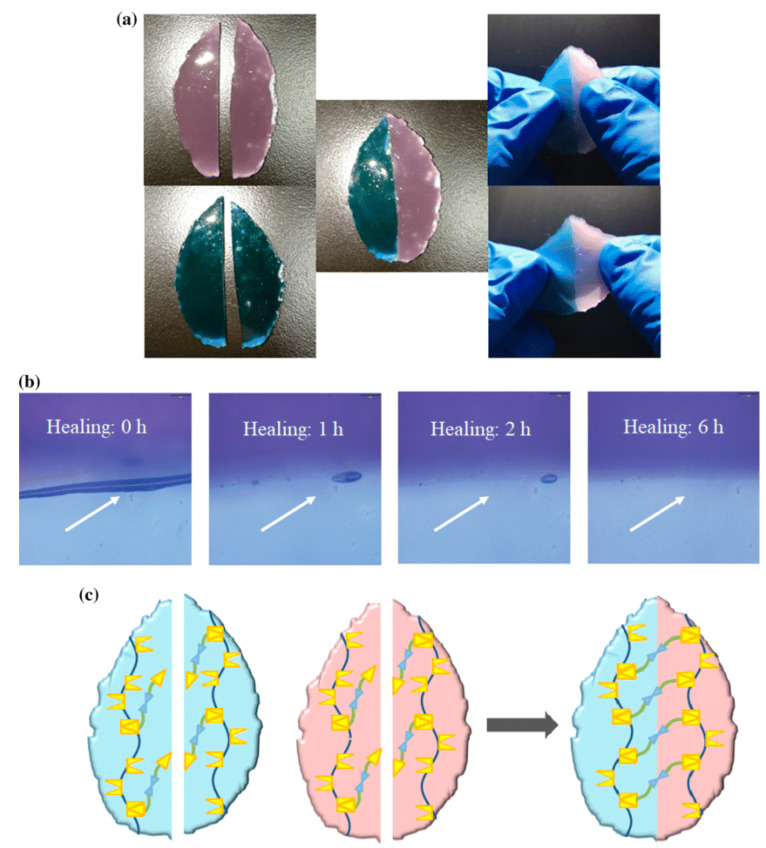
Self-Healing Hydrogels. (**a**) Macroscopic and (**b**) microscopic images of DCMC-based hydrogels (8%) pre and post self-healing, (**c**) Schematic image of the dynamic hydrogel networks through self-healing

**Figure 2:**
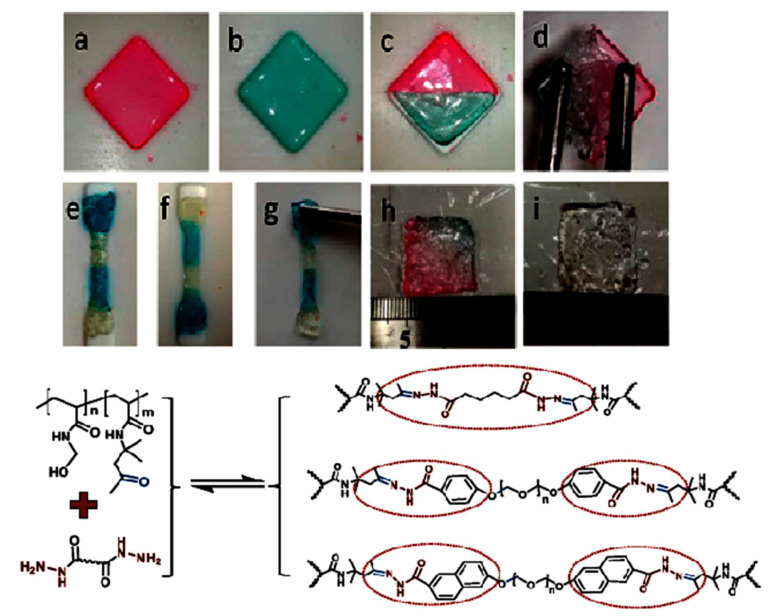
Self-healing properties of hydrogel prepared from P(AM_71_-*stat*-DAA_14_) and PEO_23_ DH cross-linking (**a**–**d**), the reshaping process (**e**–**g**), and the swell property in pH 7.4 buffer (**h**,**i**) (**upper**). Cross-linking led to a self-healing hydrogel that responds to temperature and has a gel-sol–gel transition. It is made up of dynamic covalent bonds (**lower**).

Regarding ready-mixed concrete (RMX), studies from Europe and Asia have analyzed conventional concrete and concrete environmental improvements by adding waste, recycled components, using fly ash, slag, or combining these options [10], resulting in Global Warming Potential reductions of between 1 and 23% in similar, characteristic, compressive strength. For North America, South America, and South Africa, life cycle inventories [11] and life cycle assessments [12] for conventional concrete with characteristic compressive strengths produced results between 20 and 50 MPa. Cemented paste backfill (CPB) is a unique method of mine backfilling with a potential to reduce tailings’ environmental impact [10], returning them to the underground roadway, and improving the productivity [13], safety, and efficiency of the operational procedure. It has the potential to address the safety and environmental issues that have arisen as a result of many tailings stacking. Because of the exponential increase in demand for natural resources in many countries, there is an increasing need for environmental protection at mining sites. Significant advancements have been made in both the technical and operational levels of mining equipment, as well as the technological level of the cementing agents (CA), resulting in cement backfilling mining being recognized as a contemporary mining technique that offers increased efficacy, recovery and strength rates. The commonly used cement backfilling is Portland Cement (CA) [14]. In regards to production material numbers accounted for by Chinese backfilling mines, the cemented backfilling accounts for nearly 25–40% of the overall mining expenses, and the price of CA is approximately 70–80% of the backfilling costs [15]. To lower the costs of backfilling and establish application prospects of backfilling mining technology, it is necessary to adjust to the local circumstances, acquire materials locally, and produce acceptable cement alternative materials. Around the globe today, there is a lot of effort being put into the development and implementation of cement alternative materials such as quickly cooled smelting slag, for instance: granulated blast furnace slag (GBFS) [16], steel slag [17], copper-nickel slag [18], and lead-zinc slag [19] which is a type of solid industrialized refuse due to its specific cementing activity and pozzolanic performance and has good potential for the preparation of cementitious elements via mechanical grinding as well as chemical activation. Grinding and a tiny amount of activator are all that is required to utilize these slags in cement manufacturing. It can conserve a huge ratio of energy compared to the traditional “two grinding and one burning” of Portland cement. The energy required for water-granulated slag grinding is 10% of that required for Portland cement manufacturing [9,20]. One such very promising material seems to be self-healing hydrogels, which allowed the alkali-activated principle to be identified in the 1940s due to the investigation of the chemical activation of smelting slag [21]; however, the actual analysis started in the 1960s [22]. It is necessary to raise the CaO concentration of copper slag in order to boost its cementing activity. When using NaOH as an activator, an acceptable cementing performance should be shown by a CaO mass fraction of 19 percent [23]. Figure 1 shows Self-Healing Hydrogels: (a) macroscopic and (b) microscopic images of DCMC-based hydrogels (8%) pre and post self-healing; (c) schematic image of the dynamic hydrogel networks through self-healing. Figure 2 indicates Self-Healing Hydrogels with both undergoing sol-gel transition and lower critical solution temperature (LCST) through a Cross-Linking Induced Thermo-Response.

CPB permits possibly dangerous substances to be placed into mined-out underground apertures. For the control of mineral processing tailings, CPB is an efficient and sustainable approach. Furthermore, CPB has a number of economical, operational as well as environmental benefits [24,25]. The complete gradation processing tailings (full tailings, FT), water and binders are all included in CPB. Starting from the mixing phase through the end of service life underground, each element has a significant effect on the quality and efficiency of CPB [26]. To make sure that CPB is in the correct consistency to allow for its transportation, it comprises of a lot of water [27]; this additional water can be drained when deslimed tailings (DT) are utilized. As a result, CPB strength improvements are said to be greater when DT is utilized than when FT is employed, because the latter provides for a denser cementitious matrix as well as microstructure [28]. The impact of desliming on the durability and strength of CPB was studied [26]. Nevertheless, Ercikdi et al. pointed out that desliming could have an impact on unit activities such as transporting CPB into underground voids [28]. As a result, the workability of CPB is extremely important when utilizing DT, as well as geotechnical and mechanical features. However, alkali-activated slags (AAS) have been observed to produce workability deficits in fresh concrete/mortar samples [29], even if this concern can be mitigated by adding pozzolanic ultrafine minerals or retarders. Building technologies have been adopted by man for many years and are now being investigated for contemporary buildings since they may give reduced carbon and embodied energy replacements to cement-based materials or burned masonry [30]. The lack of understanding of these materials’ mechanical qualities is a major impediment to their widespread use in modern buildings, particularly in temperate areas such as northern Europe. Modern earthy products frequently employ stabilizers, such as cement, to promote bonding, but this contradicts their environmental credentials, hence extra stabilizers are in high demand [31]. Biopolymers are polymers that are found naturally, usually from plant sources that were used as viscosifiers in the past [31]. Researchers started exploring the use of biopolymers as soil stabilizers which has been noted to reduce the permeability of the soil when put into soil biopolymers [32], boost the shear strength [33], and improve the durability [34,35]. Biopolymer stabilization alters soil characteristics by forming hydrogels during hydration. When polymer chains dry, water molecules tend to permeate, resulting in the creation of complexes of connected polymer chains. When combined with soil, hydrogels produce hydrogen and/or ionic connections between the soil particles and free water, based on the intrinsic characteristics of the biopolymer employed [36]. It can be deduced that biopolymers have a possibility of functioning as stabilizers in soil-based construction substances, giving an extra shear strength and stiffness and lacking the carbon footprint of a cement stabilizer. Due to its cost effectiveness and high compressive strength, concrete is a common medium for building material [37]. However, concrete’s susceptibility to fracture formation as a result of its low tensile strength is one of its downsides and because of that concrete is frequently blended with steel reinforcement to withstand the tensile stresses [38]. Even though these rebars reduce fracture width, they are seldom built to eliminate crack development totally. Cracks put the lifespan of concrete buildings at risk because hostile liquids and gases might permeate into the matrix and result in damage [39]. As a result, fractures may deepen and reinforcement might be exposed to the surroundings and when the cement begins to corrode, the building may collapse. Therefore, it appears self-evident that concrete crack monitoring, maintenance, and repair are all necessary. However, when the fractures are not evident or approachable, crack healing becomes harder. Concrete and reinforced concrete self-healing, also known as autogenous healing, was investigated extensively [40,41,42]. Many empirical results and real-world tests have shown that concrete cracks may heal themselves and the water flow through cracks can be controlled in time. Additionally, in Europe, healing costs account for half of the annual construction costs [43]. Besides the direct costs, secondary charges such as loss of production and traffic have a huge economic effect. That is why self-healing of cracked concrete would be incredibly helpful. Although autogenous healing is confined to tiny fractures, it works only when water is available, and is tough to regulate. However, concrete may be changed to incorporate self-healing fissures. A major conceptual principle in operations such as design, manufacturing, building, maintenance, management, repair, and destruction of buildings or any civil engineering activity that forms the environment is the sustainability approach. As a result, sustainability must be included in performance-based design and evaluation, and it has been at the forefront of research agendas across the world in recent years. Fib Commissions 7 and 10 are working on the new Model Code 2020, with sustainability as one of the major advancement goals that would be used for subsidiary function needs critical to structural design, reliability, integrating the life cycle perspective, end-of-service-life issues, and performance-based concepts [44,45]. Engineers and stakeholders must clearly examine the combined effect of the service life and applicable safety level of structures on economic and environmental issues while working with concrete [46].

### 1.1. Autogenous Healing

**Figure 3:**
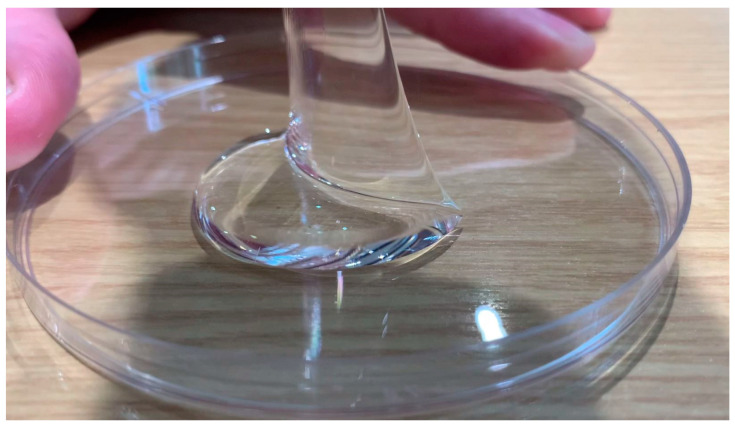
Smart hydrogel and its application in medical issues.

Autogenous healing is a well-studied procedure of internal crack healing in cement-based materials [47]. The main processes for autogenous fracture healing are, firstly, the hydration of unhydrated cement particles and, secondly, the dissolution and consequent carbonation of Ca(OH)_2_ [48]. Aside from these methods, autogenous healing can also be caused by matrix swelling and crack plugging because of debris in the loose concrete particles arising from cracking. However, the contributions of these processes are up for debate. The process with the greatest potential for autogenous healing appears to be dependent on the concrete age in cracking. In fresh concrete, continuing hydration is the principal healing method given the comparatively higher percentage of unhydrated cement particles. Calcium carbonate precipitation becomes the dominant mechanism as it ages [47]. While there are differing viewpoints on the basic process underlying autogenous healing, researchers concur that water is required for each method. The maximal crack width that could be healed by autogenous healing varies significantly between findings by different researchers, ranging from 5 to 10 µm, 100 µm, 200 µm, 205 µm, and 300 µm. They looked at the influence of crack closure on autogenous healing efficacy and found that using compressive forces to make both of the crack faces contact each other boosted healing. Figure 3 shows smart hydrogel and its application to medical issues. Concrete, in general, has the ability to heal fractures on its own. The precipitation of Calcite is created by the interaction of $CO_{3}^{2-}$ in the water supplied into the crack and ${Ca}^{2+}$ ions are dissolved in the paste. Thus, a subsequent hydration reaction in the fracture is the major source of autogenous healing [49,50]. The self-healing characteristics of concrete differ based on the kind of hydration level and binder used over time as well as the unreacted cement ratio clinker present in the mix. Because the cement granules are not fully hydrated as they become larger, they are more likely to be unreacted. The early strength of cement was recently thought to be significant. Therefore, the cement grains are finer than before. As a result, autogenous healing performance is reduced [51]. The cracked self-healing capability of cement composites using fly ash (FA) has been documented by several studies [52]. Jaroenratanapirom et al. used a crack-closing test to assess the self-healing abilities of silica fume, crystalline admixture and FA, finding that if the fracture width was greater than 0.25 mm, the findings were effective.

Since a conduit for dangerous particles dissolved in fluids and gasses is created, the existence of cracks compromises the longevity of concrete and can result in the corrosion of the reinforcment [53]. Over the last ten years, a growing desire for self-healing has resulted in more study focused on correcting the damage enhancement. Self-healing materials could rectify the progression of damage and extend the longevity as well as the soundness of the cement. Concrete can heal any damage but only to an extent. Autogenous healing refers to concrete’s inherent ability to self-heal minor cracks as a result of its structure. Autogenous healing happens via the precipitation of calcium carbonate and the hydration of unhydrated cement particles within the cracks, allowing the cracks to seal and heal by themselves. The potential of concrete to mend its cracks naturally and intrinsically was initially recognized by the French Academy of Science in 1836 [54]. Edvardsen investigated self-healing in water-pressure-loaded structures, concluding that calcium carbonate has a major part in the main healing process. A decline in water permeability in the fractured concrete samples because of autogenous crack healing was observed [40]. Since unhydrated cement particles exist in a hardened concrete matrix, the hydration of cement is maintained if water seeps into the fissures. As per Granger et al., hydration of unhydrated cement results in the formation of strong new calcium-silicate-hydrates (C-S-H) crystals, which fills minor fissures [55]. Autogenous crack filling through the precipitation of CaCO_3_ has been investigated by energy-dispersive X-ray spectroscopy via epech (2006) [56].

A paper published in *Nature* by White et al. in 2001 studied the self-healing of polymer-based materials after which much effort was put into the implementation of self-healing characteristics within cementitious materials [57]. Since the cracks were inaccessible, the effective manual crack repair strategy may be challenging; so, the use of self-healing materials to induce self-healing or to enhance healing can be a possible method of addressing these challenges. The ability of these materials to self-repair cracks in the absence of external restoration or human intervention is a crucial feature.

### 1.2. Mechanism of Self-Healing due to Further Hydration of Un-Hydrated Cement Particles

In the scenario in which the concrete has cracked, unhydrated cement particles are present on the crack surface as well as within the paste matrix. In the case of unhydrated cement particles on the crack surfaces, when they are exposed to extra water, the clinker phases of the cement will dissolve. After that, ions such as calcium, OH^−^, and silicate start diffusing into the extra water present in the cracks. This diffusion in the crack can lead to a rise in the ion content and results in the production of new hydration products within the crack once the super saturation standards have been achieved. Furthermore, once the cement paste is exposed to water, a few ions from unhydrated cement within the cement paste begin diffusing into the crack solution. Ions collect in the crack’s solution until a significant amount of super saturation is reached, causing precipitation. The drainage of bulk cement paste promotes the self-healing of cracks, in addition to the increased hydration of the cement particles [58]. The fact that portlandite is the most soluble hydrate is well recognized. When the ion content in water is less than the ion content in the bulk cement paste, ions will leach from the bulk cement paste into the cracks [59].

### 1.3. Supplementary Cementitious Materials (Slag and Fly Ash)

Ground granulated blast furnace slag has been utilized as a supplemental cementitious material for over a century. In Germany in 1888, slag and Portland cement were combined for the very first time, with bituminous coal (coal or fly ash) having been discovered more recently. After WWII, the combustion of powdered coal had been introduced. Slag and fly ash are now widely used in concrete as a component of the cement or as a supplement (mineral additive). The use of slag and fly ash reduce concrete production costs, as well as improve the longevity of the material. Fly ash and slag are synthesized as waste materials or by-products. Slag and fly ash respond much slower than cement clinkers, as per the literature [60], hence a considerable percentage of slag and fly ash stays unreacted in slag cement paste despite casting for many years. In slag and fly ash cement pastes, further reactivity of these unreacted particles creates the potential for self-healing [61]. Once it is activated, the blast furnace slag dissolves, whereas the Si-O-Si bonds in fly ash have to be broken thus resulting in it decomposing but not dissolving. Calcium silicate hydrates are formed when the disintegrated residues of the fly ash interact with lime created by Portland clinker hydration and water. Slag just needs to be activated (hydraulically latent), whereas fly ash also requires lime (pozzolanic). Slag activates at a pH of around 12, but fly ash requires a pH greater than 13 [62]. It should be emphasized that the presence of calcium hydroxide is required for the subsequent interaction of fly ash and slag during the healing process [63]. Calcium hydroxide is insufficient for more reaction of fly ash and slag when a substantial amount of cement is substituted with slag and fly ash. This creates a negative effect on the effectiveness of self-healing. Despite the fact that linear theory provides a variety of tools and methodologies, challenges surrounding the analysis of nonlinear systems have prompted scientists to discover new ways of compactly conveying information. The use of SVMs is a solution among other options, such as fuzzy logic, NNs, or genetic programming, which normally suffer from the presence of multiple local minima, the structure selection (hidden layer number or node/rule number, population size) problem, and overfitting because of their powerful regression and classification abilities according to numerical findings. The mapping of the input vector to a feature space, where the regression may be conducted much more efficiently, is a common property of ANFIS and SVMs. The main distinction in SVM-based learning is that the upper limit of a cost function is minimized rather than the cost itself. In light of each mode’s performance and shortcomings, its integration might be a viable solution to nonlinear issues [64].

### 1.4. Objectives and Problem Statements

In recent times, hydrogels have received much interest as a self-healing agent. The goal is to see the effects of the chemical makeup of hydrogels and how they behave in cementitious materials. Understanding how hydrogel activity, including the absorption in cementitious materials, affects the properties and microstructure of cement-based materials requires knowledge of hydrogel behavior. Despite the known chemical interactions between hydrogels and pore solution, this article intends to fill a gap in knowledge in comprehending the elements that affect hydrogel absorption in cement mixes.

Thus, the impact of hydrogels with nano silica (*NSi*) on compressive strength, autogenous shrinkage and the electrical resistivity of cement-soil pastes (slag, fly ash), as well as the study of the self-healing properties of cementitious materials, have been analyzed here. All test results were analyzed by use of a hybridized soft computing model such as ANFIS and SVR for precise study and to avoid few empirical tests or error percentages.

### 1.5. Significance of Study

The core concepts of materials utilized in hydrogels, such as synthetic and natural polymers, synthesis methods and varieties of hydrogels based on crosslinking methods, all have been thoroughly covered in this paper. Furthermore, the features of hydrogels, such as swellability, biodegradability, and biocompatibility of mechanical strength have been demonstrated using modern examples of hydrogels from the main to the most recent notions. Finally, hydrogels as electrolytes are discussed, as well as their self-healing and mechanical capabilities and their uses in electrochemical and biomedical devices. The use of a hybridized ANFIS and SVM algorithm in hydrogel investigations is a first for this research.

## 2. Methodology

### 2.1. Material

The German Collection of Microorganisms and Cell Cultures provided a pure culture of *Bacillus pseudofirmus* DSM 8715 (DSMZ). *B. pseudofirmus* DSM 8715 is a lake-bed soil-isolated *alkalophilic* aerobe. According to DSMZ, the ideal development temperature is 30 °C and pH is 9.7; *B. pseudofirmus* thrives in a pH range of 7.5 to 11.5 and has been shown to be resistant to oxidative damage in very alkaline environments. For later usage, a *B. pseudofirmus* overnight culture was kept in 25 percent (*v*/*v*) glycerol at 80 °C. For *CaAlg* capsule manufacturing, sodium alginate (FMC Biopolymer, *Manugel* GHB, Philadelphia, PA, USA) with a monomeric content of 37 percent *mannuronic acid* (M) and 63 percent *guluronic acid* (G) was acquired. In all series, four specimens of 25 × 75 × 75 [mm] were prepared. The cement paste and mortars were made with ordinary Portland cement (Type I). Table 1 shows the NSi content percentage. Table 2 shows the mix design and flowability of cement pastes hydrogels containing nano-silica particles for internal curing study.

### 2.2. Hydrogel

The hydrogels were produced by Ghent University’s Polymer Chemistry and Biomaterials Group (*PBM*-*UGent*) (*PBM*-*UGent*). When UV irradiation was employed to crosslink the *Pluronic F-127 bis* methacrylate (*Pluronic*-*BMA*) in the presence of the initiator (*Irgacure* 2959), the double bonds made it feasible. *Irgacure* 2959 then produces free radicals, which engage with the methacrylate moieties to initiate the *polymerization* process. Propagation, termination and initiation are all elements of the crosslinking process.

### 2.3. Encapsulation of Bio-Reagents into the Hydrogel (HA)

The word “bio-reagents” in this work makes reference to the nutrition for bacteria (yeast extract) as well as the deposition agents (calcium-nitrate, urea). To start, the bio reagent powders were added to the polymer solution. The initiator was introduced after they had fully dissolved in the polymer solution. Following that, the entire mixture was degassed followed by the identical methods as stated above for manufacturing pure hydrogel sheets. For each 10 mL hydrogel sheet, 0.4 g yeast extract, 0.9 g urea, and 1.2 g Ca(NO_3_)_2_4H_2_O were utilized. The bio-reagent-loaded hydrogel was designated by the letters HA.

### 2.4. Adaptive Network Fuzzy Inference System (ANFIS)

**Figure 4:**
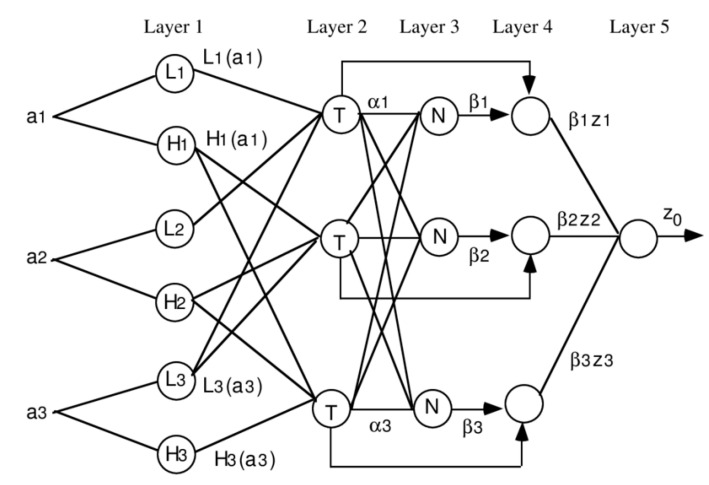
ANFIS model and its nodes.

Traditional mathematical techniques, including differential equations, are not well-adapted for dealing with ill-defined and unexpected systems in system modelling. A fuzzy inference system utilizing fuzzy if–then rules, on the other hand, may simulate qualitative components of human knowledge and reasoning processes without depending on correct numerical analysis. Takagi and Sugeno were the first to investigate fuzzy modeling or fuzzy identification in depth [65]. Conversely, there are several fundamental characteristics of this methodology that need to be clarified: 1. There are no standard ways for converting human experience or knowledge into a fuzzy inference system’s rule base and database; 2. Reliable ways for modifying membership functions (MFs) to decrease output error measure or increase performance index are needed. The fundamentals of fuzzy if–then rules and fuzzy inference systems are described in the following section. The ANFIS architecture is developed by integrating the fuzzy inference system into the framework of adaptive networks. An adaptive network, as illustrated in Figure 4, is a multi-layer feedforward network where every node performs specifically on incoming signals and a set of node-specific variables.

The nature of node functions varies between nodes, and the selection of each node function is determined by the overall input–output function that the adaptive network must perform. There are parameters on a square node/adaptive node, but none on a circular node/fixed node. These variables are adjusted depending on the gradient-based learning approach and training data detailed below to reach the appropriate input–output mapping.

### 2.5. Developing of Support Vector Machine (SVM)

**Figure 5:**
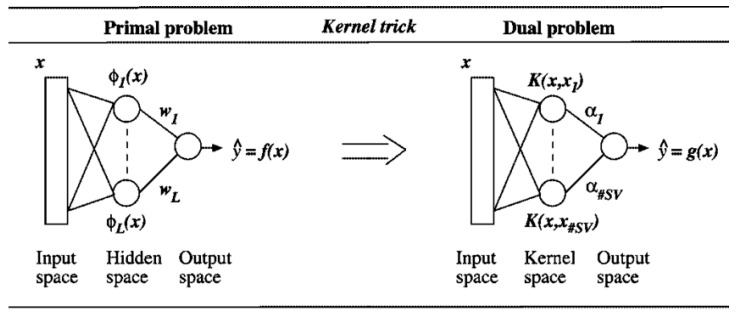
SVM model.

The regression flaws are classified using SVM as a learning technique. When considering the training pattern sets, each of which belongs to one of two groups, new instances are assigned to one of two SVM training methods. As a result, SVR aims to achieve a performance that is as close to the real target vectors as possible for all of the provided training data. SVR is assigned to kernel functions (k) graphing the data to feature space, whereby it has been popularized as the kernel trick due to the fact that linear regression in feature space indicates a non-linear regression in the original issue space. Few kernel factors are determined for this reason, although the radial basis function (RBF) kernel and the polynomial (Poly) function kernel often produce good results in comparison to other kernels (Figure 5).

## 3. Results and Discussions

**Figure 6:**
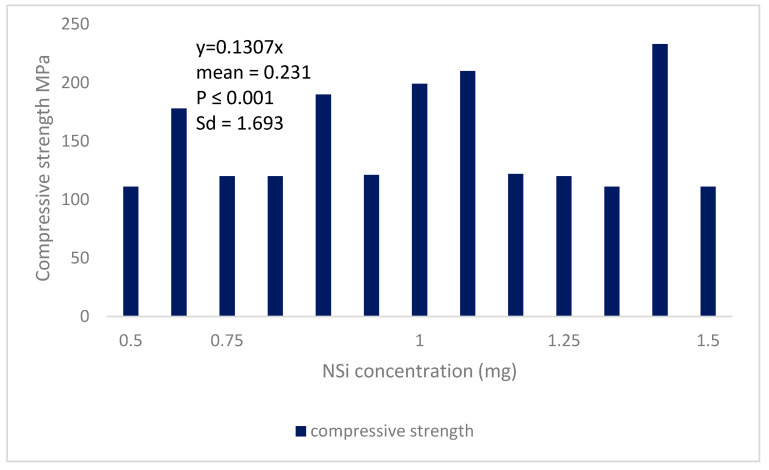
Compressive strength decrement in the cement-soil pastes by the raise of NSi concentration.

**Figure 7:**
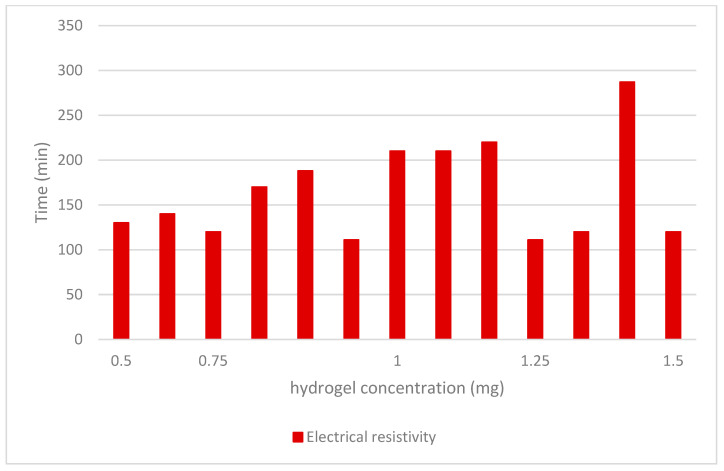
Electrical resistivity in the cement-soil pastes by the increase of NSi concentration.

**Figure 8:**
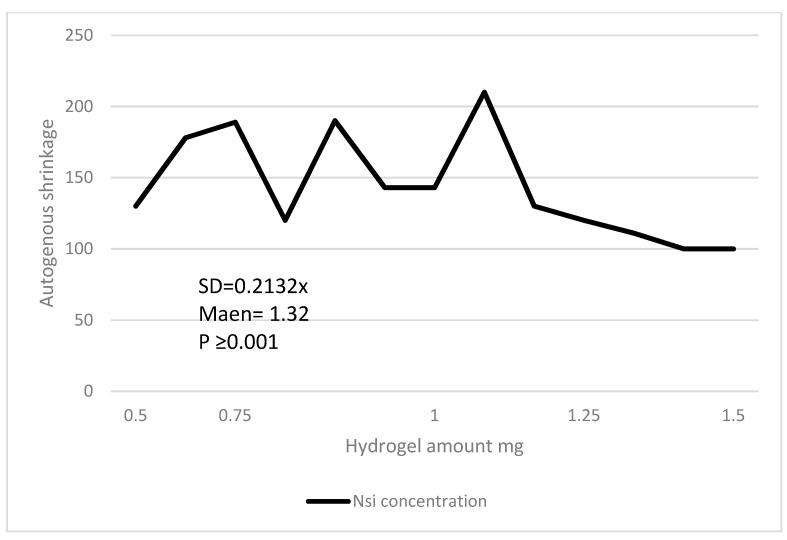
Decrement in autogenous shrinkage of cement-soil pastes while adding hydrogel.

ANFIS and SVM are two alternative data analysis strategies that were investigated in this study. The design of models and their outputs will be covered in the following sections. Figure 6 shows a compressive strength decrement in the cement-soil pastes by the increase of NSi concentration. Figure 7 shows electrical resistivity in the cement-soil pastes by the increase of NSi concentration. Figure 8 shows a decrement in the autogenous shrinkage of cement-soil pastes while adding hydrogel.

### 3.1. Compressibility and General Stress–Strain Behavior

Modeling the changing stiffness of a material undergoing cementation is difficult because the behavior of an un-cemented material progressively transforms to that of a completely cemented material during the hydration process. As a result, at all phases of the processing with the development of ultimate strength and initial yield stress as well as the development of small-strain stiffness, it is required to account for the material’s whole compressive stress–strain response. A crucial point is that stiffness, yield stress, and ultimate strength all change at the same ratio, and that the ratio of development of any one of these variables may be utilized to infer the rate of evolution of the others. The proposed method was used to simulate a series of one-dimensional compression tests on cemented CSA hydraulic fill, in which the samples were prepared at various densities and left to cure for different times before the loading to show its validity in modeling compressibility. The material constants d, X, W, and Z in Equation (1) were defined from the measured parameters of $q_{u}$ and from $G_{0\;}$gained from bender elements. The parameters λ* and k* in Equation (2) were defined from one-dimensional compression tests on uncemented material, and via changing terms A in Equation (1) and b in Equation (2). This suggests that, given a sufficient set of empirical findings, the suggested constitutive relationship accurately describes the behavior of materials compression, undergoing curing and cementation breakdown.

(1) $$dHyd=\left[\exp\left(\frac{-d}{\sqrt{t^{*}+\Delta t}}\right)-\exp\left(\frac{-d}{\sqrt{t^{*}}}\right)\right]A\exp\left(\frac{X\cdot C_{c}+C_{c}^{0.1}-e}{Z\cdot C_{c}+W}\right)$$

(2) $$dD=\frac{\left(1+e\right)bp_{y}^{\prime }\left|d\varepsilon_{v}^{p}\right|}{\left(\lambda^{*}-\kappa^{*}\right)\left\{1+b\left[\ln\left(\begin{array}{c} p_{y}^{\prime }\\ p_{o}^{\prime } \end{array}\right)-c\right]\right\}}\left[\ln\left(\frac{p_{y}^{\prime }}{p_{o}^{\prime }}\right)-c\right]$$

### 3.2. Elasticity

A 2000 kN servo test equipment was used to determine the compressive strength of the required age sample. However, the DH 3818 acquisition instrument recorded the damage. The elastic modulus and compressive strength were computed by applying the GB/T 50081-2019 code as a guide. Based on CECS 21:2000, the GTJ-U820 ultrasonic instrument was used to estimate ultrasonic pulse velocity. The elastic modulus of the specimens with a w/c ratio of 0.55 could be found to range from 24.08 to 29.00 GPa, with an 18.23% variance range. The elastic modulus of the full replacement of hydrogel for coarse aggregate (CG100MK0) is 24.08 GPa, which is 10.67 percent lower than the control sample. The samples including 10% fly ash and 0% GG with a 0.55 w/c rate had the maximum elasticity modulus, which is 29.00 GPa. Furthermore, for the specimen with a w/c ratio of 0.45, the elastic modulus varies from 26.50 GPa to 33.02 GPa with a fluctuation range of roughly 24.59 percent. The elastic modulus of the sample with 100 percent lowers to 18.03 percent in comparison to the control sample. Furthermore, the sample containing 10% fly ash and slag shows just a 2.13 percent increment in the elastic modulus. Except for the uncracked samples, the coefficient of water permeability declined for all samples until 3 days. Nevertheless, the ratio of decrement in the coefficient of water permeability in all samples decreased dramatically after 3 days. The physic mechanical, workability, and geotechnical characteristics of soil-cementitious hydrogel are all influenced by these elements. The inclusion of hydrogel, on the other hand, leads to a considerable reduction in elastic modulus that is linked to the low rigidity. The structural experiment on cement pastes, comprising of hydrogels, had favorable results [66]. It was expected that since most acrylic acid hydrogels had negatively charged groups, they would absorb more calcium ions. In line with this, Wang et al. discovered that the achieved hydrogels had superior compressive strength, a quick self-healing process (10 min), a high healing performance (over 90%), agile pH-driven shape memory behavior (recovered in 260 s), and Ca^2+^ driven shape memory behavior [67]. Cihangir and Akyol investigated the influence of tailings desliming on the hardened and workability characteristics of sulfide-rich tailings cemented paste backfill (CPB) incorporating alkali-activated slags. The water-retention capabilities of full (FT) and deslimed tailings (DT), as well as the workability features of CPB mixes, have been investigated in this context. DT was discovered to reduce water retention capacity, resulting in denser CPB combinations. Furthermore, the use of DT-AASs had no negative impact on the workability of fresh CPB materials. Because of its dispersing action, LSS-S (slag activated with liquid sodium silicate) increased the flowability of CPB mixes [68]. Water-retention capacity is significantly influenced by the mineral composition and particle size distribution in cases of clay minerals and fine particles as mentioned above, respectively.

### 3.3. Model Performance Indicators

In order to comprehend the topic, it is important to thoroughly assess the performance of the model. Eighty percent of the data are completely separated for the training part while 20% are designated for the testing part. Statistical model performance indicators such as the root square root error (RMSE), determination coefficient (R^2^) and Pearson correlation coefficient (r), were employed.

(3) $$r=\frac{N\left(\sum_{i=1}^{N}O_{i}.P_{i}\right)-\left(\sum_{i=1}^{N}O_{i}\right).\left(\sum_{i=1}^{N}P_{i}\right)}{\sqrt{\left(N\sum_{i=1}^{N}O_{i}^{2}-{\left(\sum_{i=1}^{N}O_{i}\right)}^{2}).(N\sum_{i=1}^{N}P_{i}^{2}-{\left(\sum_{i=1}^{N}P_{i}\right)}^{2}\right)}}\;$$

(4) $$R^{2}=\frac{{\left[\sum_{i=1}^{N}\left(O_{i}-\bar{O}\right).\left(P_{i}-\bar{P}\right)\right]}^{2}}{\sum_{i=1}^{N}\left(O_{i}-\bar{O}\right).\sum_{i=1}^{N}\left(P_{i}-\bar{P}\right)}\;\;$$

(5) $$RMSE=\sqrt{\sum_{i=1}^{N}\frac{1}{N}{\left(O_{i}-P_{i}\right)}^{2\;}}.$$

N = the number of training or testing samples

$O_{i}$ = observed values in sample i

$P_{i}$ = predicted values in sample i

$\underline{O}$ = the mean observed values

$\underline{P}$ = the mean predicted values

### 3.4. Discussion Analysis 

**Figure 9:**
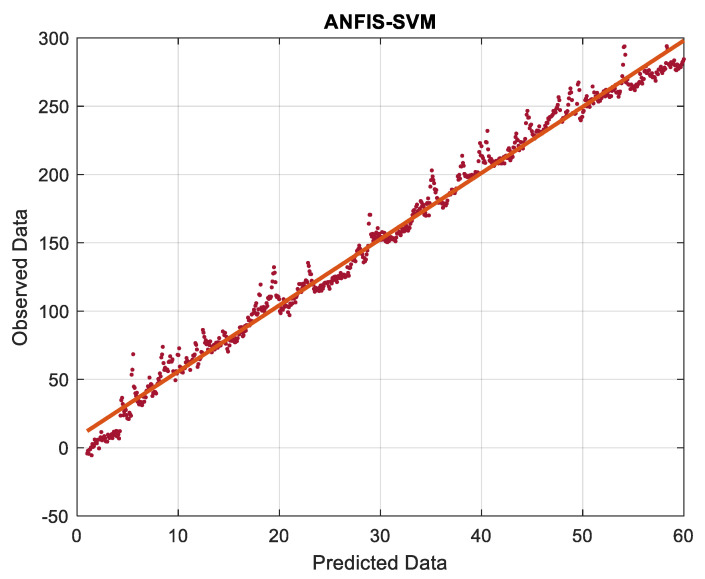
Data distribution of model.

**Figure 10:**
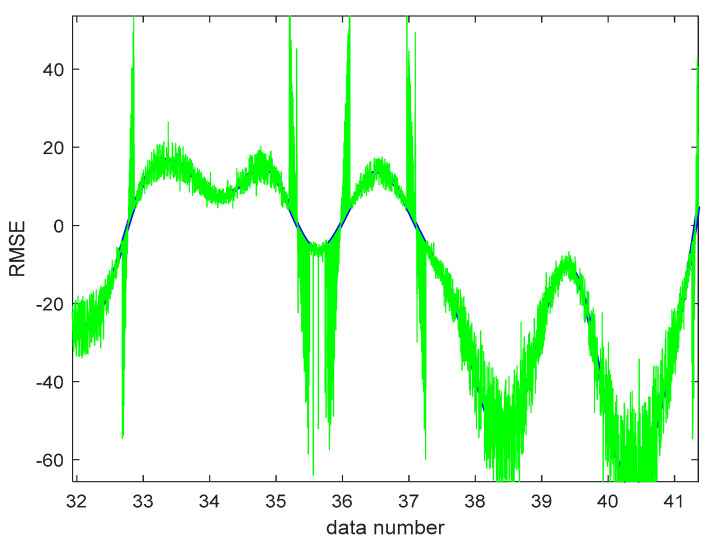
RMSE of the ANFIS-SVM.

The function of the involved parameters in the ANFIS and SVM models in terms of previously measured performance metrics was assessed in the training and testing stages after they were analyzed. Going through the Figure 9 and Figure 10, there is a good correlation between the regression line and red dots and green waves, respectively. The more dots that are over the line, the more accurately the model works (Figure 9 and Figure 10). By analyzing the major parameter to analyze the performance of the two models with regard to prediction capabilities, the goodness of fit modeled the testing phase of the data. The RMSE values of the two models were set aside to draw comparison among the regression parameters, and the model with the value nearest to zero had the greatest performance. Correspondingly, the best RMSE of ANFIS is 0.6568 in the training phase, and the best RMSE of SVM is 1.2564 in the training phase. Looking at the RMSE of ANFIS-SVM 0.5603 in the test phase, it is close to zero, showing a better performance in hypothesizing self-healing soil-cementitious hydrogel materials in mine backfill (Table 3). Comparing the hybrid model with other models as separate, the results showed that the hybridized model outperforms the individual models. If the R-square outcomes are close to one, it is the best performance for that model, thereby the RSQR of ANFIS –SVM with 0.9547 could be accepted as the proper model for predicting the study’s goal.

## 4. Conclusions

The focus of this research was to get a basic insight into the interaction between cementitious materials and hydrogels of various chemical formulations, as well as the influence of the hydrogels on cementitious materials’ self-healing behavior. This work introduced a fundamental and systematic investigational study on self-healing behaviors of cementitious materials when in the presence of hydrogels. Because of the additional hydration of unhydrated cement particles, the self-healing behavior of cement pastes with and without hydrogels was examined. The test’s hydrogel absorption measurement in the extracted pore solution may be different from the absorption assessment in cement admixture employing a hybridized soft computing model. The chemical interactions between the hydrogel surface and hydrating cement particles have been shown to play a significant role in the absorption of hydrogels in a cement admixture; the formation of a relatively stiff skin on the surface of the hydrogel because of the interactions between the hydrating cement particles and the hydrogel could reduce hydrogel absorption. The findings demonstrated that the healing rate recorded in samples incubated in sand was often lower than that observed in samples incubated in water. As maximal healing rates were reported for both sample types, these data show that water incubation is still the most suitable environment for self-healing. Except for the uncracked samples, the coefficient of water permeability declined up to 3 days. Nevertheless, after 3 days, the lowering ratio of the coefficient of water permeability in all samples decreased dramatically. These elements have a substantial impact on the soil-cementitious hydrogel’s physic mechanical, workability, and geotechnical characteristics. Moreover, hydrogel absorption can be influenced by adding extra cementitious materials; usually, a boost in hydrogel absorption was noticed when glass powders were employed. As a consequence, since this absorption depends on the chemical compositions of hydrogels, an increment in acrylic acid/acrylamide lowered the hydrogels’ absorption.

## Figures and Tables

**Table 1:** NSi content ratio.

NO.	Hydrogel	Distilled Water	AA *	AM	NaOH	MBA	Alg	APS	NSi Fine (gr)	Colloidal NSi (gr)	Water- Lass (gr)
1	H-2	45	10	10	1.35	0.05	-	0.128	-	-	-
2	H-3	43	2	15	0.27	0.05	-	0.128	-	-	-
3	H-a	54	-	30	-	0.05	0.6	0.128	-	-	-
4	0%- Reference Hydrogel	65	-	10	-	0.05	-	0.64	-	-	-
5	5%-NSi	90	-	10	-	0.04	-	0.53	1	-	-
6	10%-NSi	90	-	10	-	0.04	-	0.53	2	-	-
7	20%-NSi	100	-	10	-	0.04	-	0.53	4	-	-
8	50%-NSi	100	-	10	-	0.04	-	0.53	10	-	-
9	CNSi	20	-	10	-	0.04	-	0.53	-	50	-
10	WG	34	-	10	14	0.04	-	0.53	-	-	30

* All contents are as %.

**Table 2:** Mix design and flowability of cement pastes hydrogels containing nano-silica particles for internal curing study.

Paste Designation	Water/Binder	Superplasticizer (%)	Hydrogel (%)	Flowability (cm)
Ctrl-0.3	0.3	0.4	-	20
Ctrl-0.35	0.34	0.4	-	22
0%-Ctrl	0.34	0.4	0.3	21
C-5%-NSi	0.34	0.4	0.35	21
C-10%-NSi	0.34	0.4	0.4	21
C-20%-NSi	0.34	0.4	0.5	21
C-50%-NSi	0.34	0.4	0.7	21

**Table 3:** The regression output for self-healing soil-cementitious hydrogel materials in mine backfill in both phases.

Models	RMSE	r	R^2^	RMSE	r	R^2^
**ANFIS**	0.6568	0.960	0. 8765	0.7888	0. 9654	0.9187
**SVM**	1.2564	0.0868	0.7786	1.310	0.7654	0.9079
**ANFIS-SVM**	0.6643	0.8754	0.9311	0.5603	0.6543	0.9547

## Data Availability

Not applicable.
